# Marinacci Anastomosis: A Case Report

**DOI:** 10.7759/cureus.19034

**Published:** 2021-10-25

**Authors:** Santosh L Wakode, Naveen Ravi

**Affiliations:** 1 Physiology, All India Institute of Medical Sciences, Bhopal, IND

**Keywords:** ulnar to median communication, marinacci anastomosis, reverse martin gruber anastomosis, nerve conduction study, electrophysiology

## Abstract

Marinacci anastomosis or the reverse Martin-Gruber anastomosis is a rare anomalous intercommunication from the ulnar nerve to the median nerve in the forearm. A young lady was involved in a road traffic accident that resulted in the fracture of both forearm bones for which she was treated surgically. A nerve conduction study was performed later on, which incidentally demonstrated findings of this communication. Knowledge of this anastomosis can help prevent misdiagnosis of clinical findings and misinterpretation of electrophysiological studies and help mitigate iatrogenic injury during surgical procedures.

## Introduction

Anomalous intercommunications between the ulnar and median nerves have been described in both the forearm and hand. These intercommunications between the nerves provide a detour route for nerve fibers going to the hand [1]. Some distal function may be spared by these anomalous intercommunications, in cases of ulnar and median nerve lesions, with the detour route bypassing the block in conduction [1]. These are usually asymptomatic and are incidentally discovered during surgery, following nerve injury, during routine electrodiagnostic tests, or during routine cadaveric dissections [1-11]. The common anomalous intercommunications in the forearm and hand include the Martin-Gruber and Marinacci anastomoses in the forearm and the Riche-Cannieu and Berrettini anastomoses in the hand. In Martin Gruber anastomosis (MGA), the intercommunication is between the proximal median nerve to the distal ulnar nerve; the incidence of MGA is reported to be around 26% [4,6,12]. The reverse MGA or the Marinacci anastomosis (MA) is quite rare with an incidence of 1.3% to 16.7% [13-14]. MA typically carries motor fibers from the ulnar to the median nerve and is among the least studied forearm anastomoses [6,12]. In MA, hand muscles that are typically supplied by the median nerve, such as the abductor pollicis brevis (APB), get innervated by the ulnar nerve [6,12]. We present a clinically suspected case, which was subsequently evaluated using electrophysiological studies.

## Case presentation

A lady in her mid-20s was involved in a road traffic accident during which her right forearm came under the wheels of a car. She was rushed to a nearby hospital where radiographs revealed a closed fracture of the shafts of both forearm bones. She subsequently underwent open reduction and internal fixation + primary bone grafting of the radius and ulna. The bone graft was harvested from the ipsilateral olecranon. About six weeks post-surgery, when her plaster slab was removed, she noticed the inability to flex her right thumb and right index finger. When the stiffness in her fingers persisted even after a couple of weeks, she presented to our institute for further management. On examination, she had hypoesthesia of the pulps of the index, middle, and ring fingers. She also had stiffness of the thumb and index finger with incomplete active flexion. She was started on hand therapy. Clinical findings were suggestive of possible median nerve involvement while it was expected that the ulnar nerve might be involved considering that the bone graft was harvested from the proximity of the ulnar nerve. Hence, to investigate further and to rule out anomalous nerve anastomosis, electrophysiological tests were performed.

**Figure 1:**
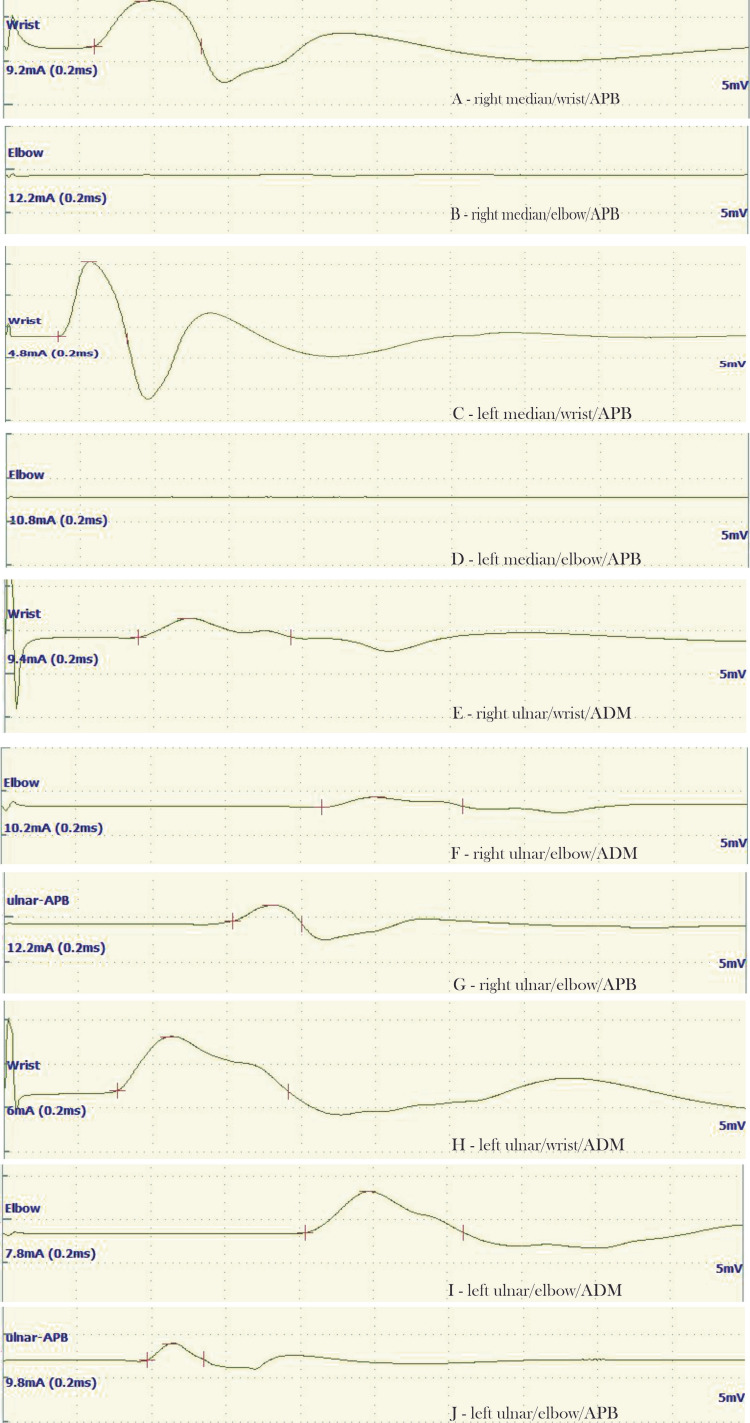
Nerve conduction study showing findings of Marinacci anastomosis A - Waveform recorded by stimulation of the right median nerve at the wrist while recording over APB; B - Waveform recorded by stimulation of the right median nerve at the elbow while recording over APB; C - Waveform recorded by stimulation of the left median nerve at the wrist while recording over APB; D - Waveform recorded by stimulation of the left median nerve at the elbow while recording over APB; E - Waveform recorded by stimulation of the right ulnar nerve at the wrist while recording over ADM; F - Waveform recorded by stimulation of the right ulnar nerve at the elbow while recording over ADM; G - Waveform recorded by stimulation of the right ulnar nerve at the wrist while recording over APB; H - Waveform recorded by stimulation of the left ulnar nerve at the wrist while recording over ADM; I - Waveform recorded by stimulation of the left ulnar nerve at the elbow while recording over ADM; J - Waveform recorded by stimulation of the left ulnar nerve at the elbow while recording over APB.

A nerve conduction study (NCS) was performed in our department using Nihon Kohden Neuropack X1 (Japan). On NCS of the right upper limb, proximal stimulation of the median nerve revealed a conduction block in the APB, whereas distal stimulation revealed normal compound muscle action potential (CMAP) in the APB (amplitude, 5.2 mV). This suggested possible involvement of the median nerve in the forearm. Proximal and distal stimulation of the ulnar nerve demonstrated diminished amplitudes at the abductor digiti minimi (ADM), which were 1.2 mV and 2.1 mV, respectively. However, proximal stimulation of the ulnar nerve elicited normal CMAP in the APB (amplitude, 1.7 mV). Sensory evaluation was equivocal. Since the presence of bilateral nerve anastomosis in the upper limb is described in the literature, we repeated NCS on the contralateral limb and recorded similar findings except for normal CMAP in the ADM (Figure 1). She was continued on hand therapy and had a complete recovery of hand function in three months’ time.

## Discussion

**Figure 2:**
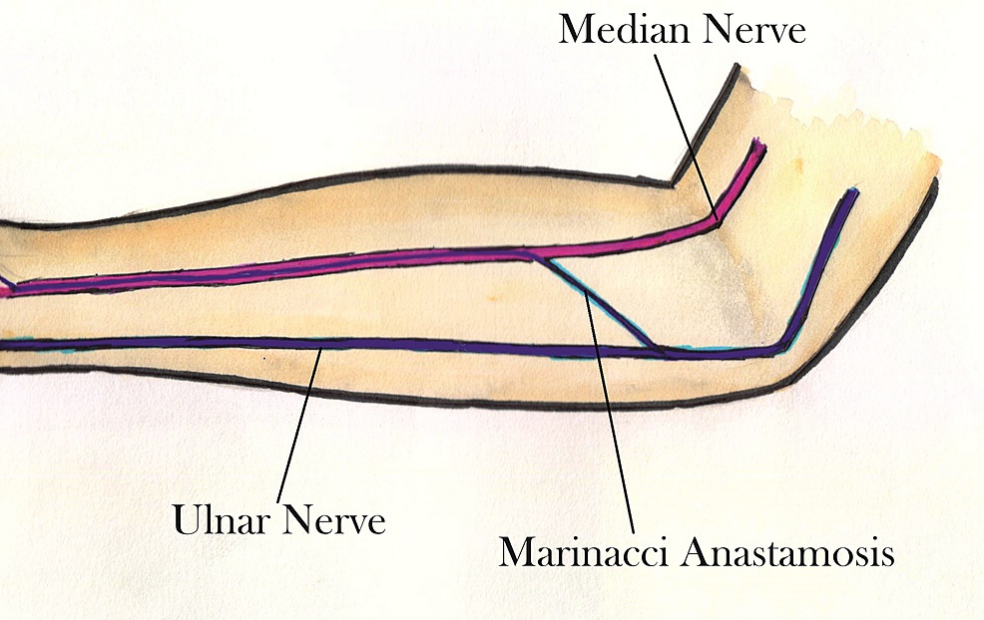
Schematic sketch of Marinacci anastamosis This figure was hand-made by both authors.

Anomalous intercommunications between the median and ulnar nerves have been extensively studied and reported. These intercommunications have been reported to occur in the brachial plexus and in the forearm and hand [6]. In 1964, Marinacci reported a patient with a median nerve injury in the forearm who had preserved function of the median innervated hand muscles despite denervation of forearm flexors. The term ‘Marinacci anastomosis,’ for ulnar-to-median anastomosis in the forearm, was introduced by Hopf to credit the first author who described this rare condition [15-16]. Hopf reported intercommunication involving only the sensory nerve fibers arising from the median nerve distally to the ulnar nerve proximally [16]. Other authors have reported a motor communicating branch from the ulnar to the median nerve [2]. Our case had findings suggestive of a motor communicating branch from the proximal ulnar nerve to the distal median nerve (Figure 2).

Golovchinsky emphasized exerting special care while evaluating the motor distal latency of the median nerve with a gradual and slow increase of the stimulus voltage in suspected ulnar to median anastomoses, as the use of high voltage from the beginning can simultaneously activate both the median nerve and a collateral branch of the ulnar nerve evoking a short-latency response in the thenar muscles with a simultaneous long-latency response to the stimulation of the median nerve being masked by this fast response [14]. Amoiridis and Vlachonikolis demonstrated that supramaximal stimulation of the ulnar nerve about 1 cm above the medial epicondyle of humerus stimulus spread to the median nerve was possible [17]. We took care of these factors while evaluating our patient.

Sundaram et al. studied 100 subjects (200 extremities) and found electrodiagnostic evidence of MA in seven limbs, a prevalence of 3.5%, and suggested that MA was quite common but underreported [5]. They cautioned against misinterpreting changes noted over the median nerve as neuropraxia if attention is not paid to the amplitude difference between the CMAP obtained on distal and proximal stimulation of the ulnar nerve. Further, in MA, ulnar nerve injury at the elbow may produce denervation changes over median innervated thenar muscles or median nerve injury at the elbow may not result in clinically significant effects in the thenar muscles. The presence of bilateral anomalous intercommunications has been variously reported in the literature. The average bilateral incidence of MGA has been reported to be around 17%, Berrettini anastomosis (BA) 48%, and Riche-Cannieu anastomosis (RCA) 31% [1,18]. While the incidence of bilateral MA is not known, this may be the first report of a bilateral MA in literature.

Our patient had a loss of function of flexor pollicis longus (FPL) and flexor digitorum profundus of the index finger (FDPI). Transient loss of FPL function is frequently observed following plate osteosynthesis of Page 3 of 10 radius fracture; this may result from a traction injury to the nerve branch to the FPL or due to extensive stripping of the muscle origin, and this may have been the possible reason for loss of FPL and FDPI function in our patient [19-20]. Traction injury to the communicating branch was the probable reason for absent CMAPs at APB on proximal stimulation of the median nerve, which recovered in due course of time. Bone graft harvest from the olecranon could also, theoretically, result in trauma to the ulnar nerve; however, there were no signs suggestive of ulnar nerve injury on clinical examination. MA is a rare anatomical variation, and the communicating branch can be diagnosed using electrophysiological studies.

## Conclusions

Awareness of these anastomoses will lead orthopedic, trauma, and hand surgeons to be vigilant during a surgical approach to the forearm and hand. Additionally, clinicians should be aware that these anastomoses can lead to atypical clinical findings. Thorough knowledge of electrophysiological findings is a prerequisite to prevent the misinterpretation of electrodiagnostic tests.
